# Medical students' views about an undergraduate curriculum in psychiatry before and after clinical placements

**DOI:** 10.1186/1472-6920-8-26

**Published:** 2008-04-25

**Authors:** Clare Oakley, Femi Oyebode

**Affiliations:** 1 Department of Psychiatry, University of Birmingham, Queen Elizabeth Psychiatric Hospital, Mindelsohn Way, Edgbaston, Birmingham, B15 2QZ, UK

## Abstract

**Background:**

It has been suggested that medical students wish to focus their learning in psychiatry on general skills that are applicable to all doctors. This study seeks to establish what aspects of psychiatry students perceive to be relevant to their future careers and what psychiatric knowledge and skills they consider to be important. It is relevant to consider whether these expectations about learning needs vary prior to and post-placement in psychiatry. To what extent these opinions should influence curriculum development needs to be assessed.

**Methods:**

A questionnaire was distributed to medical students before they commenced their psychiatry placement and after they had completed it. The questionnaire considered the relevance of psychiatry to their future careers, the relevance of particular knowledge and skills, the utility of knowledge of psychiatric specialties and the utility of different settings for learning psychiatry.

**Results:**

The students felt skills relevant to all doctors, such as assessment of suicide risk, were more important than more specialist psychiatric skills, such as the management of schizophrenia. They felt that knowledge of how psychiatric illnesses present in general practice was important and it was a useful setting in which to learn psychiatry. They thought that conditions that are commonly seen in the general hospital are important and that liaison psychiatry was useful.

**Conclusion:**

Two ways that medical students believe their teaching can be made more relevant to their future careers are highlighted in this study. Firstly, there is a need to focus on scenarios which students will commonly encounter in their initial years of employment. Secondly, psychiatry should be better integrated into the overall curriculum, with the opportunity for teaching in different settings. However, when developing curricula the need to listen to what students believe they should learn needs to be balanced against the necessity of teaching the fundamentals and principles of a speciality.

## Background

Previous research has centred on the perceived negative attitudes of medical students towards psychiatry and the implications for recruitment. It was found that encouragement from more senior doctors during a psychiatric attachment increases the number of students wanting to pursue psychiatry [1]. In addition to fostering enthusiasm among potential psychiatrists, it is critical that all future doctors have the skills and confidence to deal with people suffering from mental health problems. Half of all people with ill health in Western Europe have a mental illness and it accounts for as much suffering as all physical illnesses put together [2]. It is important that psychiatric teaching is relevant and useful to all future doctors to ensure that all patients with mental health problems receive excellent care for both their physical and mental health problems. A previous study to establish the learning priorities of medical students and psychiatrists found that there was agreement between the groups that basic psychiatric skills needed by most doctors were more important than specialized psychiatric knowledge [3].

If these general skills are prioritised by medical students it is of interest to determine whether they believe their psychiatry placements are providing them with these skills for their future careers. In the last 10 years medical school curricula have been reformed in line with the recommendations of Tomorrow's Doctors, aiming to better prepare students for their employment [4]. The hallmark of new perspectives on learning is that they place the student at the centre of a learning programme, which leads to an expectation that they are included in curriculum organization [5]. Increasingly student feedback on their teaching and placements are being sought by medical schools. However, it is more unusual for students to be explicitly involved in curriculum development and there is limited literature on this topic. There are several advantages of student involvement, including students being aware of the education that the curriculum amounts to in practice, which can differ significantly from the curriculum as planned and they also experience the 'hidden curriculum', which includes the transmission of culture and values [5]. However, there are limitations to student input into curriculum development as it is not only what the student wants (which they determine) which is important but what the student also needs (which the teacher must determine). There is a distinction between education and training for a job.

The focus of this study was to consider what student beliefs were about what the content of their psychiatry placement and the implications for the role they should have in curriculum development. It aimed to determine if these beliefs differed between students before they commenced their placement and when they had completed their placement. This will indicate whether exposure and time or experience influence students' beliefs about their educational needs. If these expectations vary how this may impact on curriculum development needs to be considered.

## Methods

The study was conducted in the academic year 2005/2006 with 4th and 5th year medical students at the University of Birmingham. Undergraduate teaching in psychiatry in Birmingham consisted of a six week general adult placement in the 4^th ^year and a two week speciality placement in the 5^th ^year. The cohort of 5^th ^year students experienced the same programme of teaching as the 4^th ^year students.

A questionnaire was devised to assess the students' opinions about the content of the undergraduate curriculum and placement in psychiatry and its relevance to their future careers. The questions were developed in consultation with psychiatrists involved in designing and delivering the undergraduate course. The questionnaire (see Additional file 1) contained statements about the students' beliefs about the relevance of psychiatry to their future careers and other branches of medicine. The relevance of particular knowledge and skills was addressed by considering their opinions about topics from the undergraduate curriculum. Knowledge of the psychiatric specialities that students may undertake placements in was enquired about. The questionnaire also considered the utility of different settings, such as general practice, for learning psychiatry. A mixture of positive and negative statements was used to mitigate the effects of a response-set on the part of the respondent. All answers were given in the form of a Likert scale. The questionnaire was piloted on a small group of 4^th ^and 5^th ^year medical students and in response some wording was adjusted to ensure clarity.

The questionnaire was distributed to all 4th year students on the first day of their psychiatry teaching and to all 5th year students in a lecture at the end of their psychiatry course. The questionnaire was distributed at these time points in order to try to establish the effect of the clinical placement itself on the opinions of students about their education in psychiatry. Ethical approval was not required for this study. All the students received written and verbal information about the study from the first author before deciding whether to participate by completing the questionnaire and responses were anonymous.

Results were analysed using Microsoft Access, Microsoft Excel and Minitab. The statistical significance of any differences between the 4^th ^and 5^th ^year students was determined using Chi-square. As multiple Chi-square tests were carried out it was necessary to correct for Type I error. Therefore, a p-value of 0.01, rather than the usual 0.05, was used for statistical significance instead of a very conservative Bonferroni correction.

## Results

341 questionnaires were given out to the 4th years and 307 were returned (90%). The total number of students in the 4^th ^year was 356, meaning that 15 did not attend the induction and so could not receive the questionnaire. This still meant that 86.2% of the year responded to the survey. 235 questionnaires were given out to the 5th years and 197 were returned (83.8%). The total number of students in the 5^th ^year was 292, with 57 not attending the lecture and so not receiving a questionnaire. This resulted in 67.5% of the year being surveyed. There was no significant difference between the 4^th ^and 5^th ^year students in terms of gender (χ^2 ^= 4.35, d.f. = 1, p > 0.01), ethnicity (χ^2 ^= 4.31, d.f. = 3, p > 0.1) or future career choice (see Table 1).

**Table 1:** Favoured future career choice

**Future career choice**	**Percentage of 4^th ^years (n = 307)**	**Percentage of 5^th ^years (n = 197)**	**p-value**
General practice	29%	31%	
Medicine	31.9%	24.4%	
Surgery	18.6%	19.8%	
Paediatrics	5.5%	8.1%	0.27
Obstetrics and gynaecology	4.2%	5.6%	
Psychiatry	2.6%	4.6%	
Anaesthetics	4.2%	1.5%	
Don't know	3.9%	5.1%	

The responses were grouped together for analysis, for example agree and strongly agree were combined, as were disagree and strongly disagree as few respondents used the 'strongly' options. More 5^th ^years (79.2%) than 4^th ^years (60.6%) disagreed with the statement that psychiatry can only be learnt in a psychiatric hospital (χ^2 ^= 19.03, d.f. = 1, p < 0.001). The responses of the students to various statements about the relevance and utility of psychiatry are shown in Table 2. 92.2% of 4^th ^years agreed that their psychiatry placement would be a valuable experience but only 71.1% of 5^th ^years agreed that it had been (χ^2 ^= 39.67, d.f. = 1, p < 0.001).

**Table 2:** Responses to statements about the relevance and utility of psychiatry

**Statement**	**Percentage of 4^th ^year students who agreed n = 307**	**Percentage of 5^th ^year students who agreed n = 197**	**p-value**
Psychiatry placement is a valuable experience	92.2%	71.1%	**<0.001**
Psychiatric knowledge is essential for my future career	84.7%	79.7%	0.15
Psychiatric knowledge is essential for a career in general practice	96.1%	98%	0.24
The presentation of psychiatric illness in general practice is important	91.5%	93.9%	0.32
General practice is a useful setting for learning psychiatry	91.5%	96.4%	0.03
The presentation of psychiatric illness in the general hospital is important	96.1%	94.9%	0.53
A medical ward is a useful setting for learning psychiatry	54.7%	60.4%	0.21

The section regarding utility of certain skills was the only section when a large proportion of respondents used the strongly agree option and so the responses were not combined with the agree option as was done elsewhere. The proportion of students who strongly agreed a skill was important is shown in Table 3. The results for both years were broadly similar but significantly more 5^th ^years felt that assessment of suicide risk and self-harm (p < 0.001), management of alcohol withdrawal (p < 0.001) and recognition and management of delirium (p < 0.001) were important than the 4^th ^years.

**Table 3:** Skills which students strongly agreed were important

**Skill**	**Percentage of 4^th ^years strongly agreeing it is important (n = 307)**	**Percentage of 5^th ^years strongly agreeing it is important (n = 197)**	**p-value**
Assessment of self-harm and suicide	40.7%	65%	**<0.001**
Management of alcohol withdrawal	37.5%	54.8%	**<0.001**
Diagnosis and management of depression	43.3%	51.8%	0.06
Recognition and management of delirium	25.4%	51.3%	**<0.001**
Assessment of substance misuse	39.1%	50.3%	0.01
Treatment of bipolar affective disorder	13.4%	13.2%	0.96
Cognitive-behavioural therapy for anxiety disorders	14.3%	9.1%	0.08
Assessment of cognitive function	36.2%	45.7%	0.03
Understanding the Mental Health Act	33.6%	32.5%	0.80
Assessment of personality disorder	25.4%	5.5%	0.01
Assessment of psychotic symptoms	19.5%	25.9%	0.09
Management of schizophrenia	17.6%	20.8%	0.36

Both the 4^th ^and 5^th ^years ranked addictions followed by old age as the most useful specialties to their future career. They also both ranked forensic as the least useful specialty. The 5^th ^year students (72.1%) were more likely than the 4^th ^year students (49.5%) to agree that knowledge of liaison psychiatry would be important to their future practice (χ^2 ^= 25.14, d.f. = 1, p < 0.001). The 5^th ^years (75.1%) were also more likely than the 4^th ^years (62.2%) to agree that neuropsychiatry was useful (χ^2 ^= 9.09, d.f. = 1, p = 0.002).

## Discussion

It is noticeable that nearly all 4^th ^years thought that their psychiatry placement would be a valuable experience but that less than three quarters of 5^th ^years agreed that it had been. This indicates that the expectation of the value of their placement was not matched by the reality. It is possible that the fourth years had unrealistic expectations about the placement which coloured their views about the actual placement. Whilst it is encouraging that the majority of 5^th ^years still felt their placement had been valuable, it is helpful to consider the possible reasons for the disparity between the 4^th ^and 5^th ^years' views.

Whilst the questionnaire focuses on the content of the curriculum the quality of teaching that the students' experienced may have impacted on their experience of the placement. They may have found it less valuable due to poor teaching or unsuitable clinical placements. However, both the 4^th ^and 5^th ^years undertook the same teaching programme and the same range of placements and it was not possible in this study to account for individual experiences of the teaching received.

It is helpful to consider what other factors may have influenced the 5th years' views about their placements other than the placements themselves. It is possible that other clinical placements they had undertaken since their initial psychiatry placement in the 4^th ^year had shaped their views. It may be that trainers in other specialities, who may have negative opinions about psychiatry, have a role in changing students' opinions about the usefulness of an undergraduate placement in psychiatry.

A potentially important difference between the 4^th ^and 5^th ^years is that the 5^th ^years are approaching employment and are more focused on the skills that they will require. They placed emphasis on skills that they will need as newly practising doctors, such as assessment of suicide risk and self-harm, management of alcohol withdrawal and recognition of delirium. These skills were ranked above skills that are more specific to psychiatrists, such as the management of bipolar affective disorder and schizophrenia. It seems likely that the 5^th ^years' increased clinical exposure and experience on medical and surgical wards, where they would have encountered these conditions, led to an increased awareness of the importance of these topics. Previous work has highlighted the need to focus on psychiatric problems in the general hospital [6]. Indeed core competences for the Foundation Years in the UK (the first two years of employment following graduation) that relate to psychiatry are understanding and applying the principles of: managing a patient following self-harm; managing a patient with an acute confusional state or psychosis [7]. Old age psychiatry and addictions were felt to be the most useful specialties and deal with issues that are relevant to the Foundation Years.

More 5^th ^years than 4^th ^years felt that psychiatry can be learnt outside a psychiatric hospital. This may represent a greater understanding by the 5^th ^years of the importance of the presentation of mental health problems in other settings, for example primary care. Indeed most students felt that general practice was a useful setting for learning psychiatry, indicating the importance of collaboration between psychiatry and primary care departments. It has been found that integrating 4 to 5 sessions in general practice into a hospital psychiatric attachment demonstrated benefits of: increasing breadth of experience, understanding the patients' experience, learning about mental illness from a GP's perspective, 'normalisation' of mental illness and increased empathy [8]. However, learning psychiatry in other settings and from doctors other than psychiatrists may adversely affect students' opinions about patients with mental health problems. Research has demonstrated negative attitudes towards mental illness amongst general practitioners and hospital medical and nursing staff [9]. Only just over half the students in the current study felt that a medical ward was a useful setting for learning psychiatry, despite over two thirds of 5^th ^years recognising the importance of liaison psychiatry. There may be limited availability of liaison placements but it has been shown that teaching liaison psychiatry to students who are attached to general medical firms, by means of clinically based seminars, has a sustained impact on their knowledge [10]. The importance that 5^th ^years placed on neuropsychiatry may reflect their perception of the importance of the interface between psychiatry and general medicine.

When considering a wish to integrate psychiatry more fully into the curriculum it is important to appreciate that most medical schools are aiming to integrate subjects within their curricula to a greater degree. Most efforts to date have been with vertical integration, which is integration of basic sciences with clinical skills and practice. The next phase is integrating clinical specialities better, for example general practice and psychiatry as suggested above. There are organisational and logistical reasons why this is difficult. However, it can be achieved with problem based learning, which is an instructional method that uses patient problems as the context of teaching problem-solving skills and where attention is drawn to the complexity of cases. It can be demonstrated how a patient may present in one setting and require treatment in a variety of different settings and with input from different specialities. To ensure the optimum outcome from these methods it is essential to ensure that the students take an holistic approach and fully consider the psychosocial aspects of the problem and not solely focus on the biomedical perspective. It may be that this is best achieved by involving psychiatrists in the design and implementation of such a programme of teaching.

It is important to consider whether the opinions of the 5^th ^years are more important than those of the 4^th ^years. It can be argued that they are closer to employment and so more aware of the skills they will need to utilize. However, this does not help to improve the perceived relevance of psychiatry to 4^th ^years, who according to this study have a lesser understanding of topics which may be useful in their early years of employment. It is also important to consider how important medical students' opinions of what is useful for them to learn are at all. It has so far been assumed that learners know what they need to know. This point may be particularly relevant when considering the undergraduate curriculum in psychiatry, compared to other medical specialities, as the stigma of mental illness and negative attitudes of students may have an impact on what they perceive to be important to learn about psychiatry. Whilst perceived relevance may increase students' enthusiasm for the learning there may be topics that they perceive to be less relevant which are nonetheless crucial to learn. This may relate specifically to specialist psychiatric knowledge, which students may not feel is relevant to them if they do not want to become psychiatrists. For example, the students did not believe that knowledge of the management of schizophrenia and bipolar affective disorder was important despite this being core knowledge for a psychiatrist and that patients with these disorders will be encountered by all doctors. There must be a balance between generalist and specialist knowledge as focusing only on generalist knowledge produces different doctors.

This study examines whether students consider that their undergraduate education in psychiatry is useful and relevant for their future careers. A previous study considered whether newly qualified doctors had the skills to deal with common psychiatric problems. The survey of house officers (the first year of employment, now replaced by Foundation Years) showed that they rarely asked questions on psychological state when admitting patients to hospital and often believed they lacked the skills to assess and treat common psychiatric problems such as depression, anxiety and alcohol misuse [11]. It is not just undergraduate training in psychiatry that could be accused of not equipping students with the skills they will need in their first years of employment. Doctors have often complained that undergraduate training provides little experience to know what to expect in the working environment. Another national survey of house officers found that only 4.3% strongly agreed and 32% agreed with the statement "My experience at medical school prepared me well for jobs I have undertaken so far" [12]. A study to examine how well students are prepared for employment showed that house officers and their consultants felt they were best prepared in areas of communication skills but less well prepared in basic clinical competencies such as treatment, prescribing and managing emergencies [13]. These studies demonstrate that there are concerns that undergraduate education does not adequately prepare doctors for their early working lives.

Whilst it is important to recognise students' views about their learning needs they have not identified themes to be important that psychiatrists would consider essential. For example, assessment of psychotic symptoms was ranked eight out of the list of twelve skills by both the 4^th ^and 5^th ^years, indicating that they may not appreciate its importance. When considering what it is important to teach we believe three principles may be used to guide decisions: topics that are common; topics that are important; topics that illustrate underlying principles. Teaching students about the assessment of psychotic symptoms allows a discussion of an underlying principle of psychiatry: phenomenology. Whilst students may not recognise the importance of this it would be considered integral to an undergraduate psychiatry curriculum. It may also be that concepts such as this excite students about psychiatry as a discipline. Whilst recognising the importance of making undergraduate training in psychiatry relevant for all doctors, there is still a need to attract students to a career in psychiatry. It has been recognised for many years that there are insufficient United Kingdom graduates interested in pursuing a career in psychiatry to meet the demand for psychiatrists [14]. There were more 5th years than 4th years intending to pursue a career in psychiatry (see Table 1) but this was not statistically significant. Overall, 3.4% of students were intending to pursue a career in psychiatry, which concurs with the recent finding of 3.6% of newly qualified doctors [15]. It is possible that a student's enthusiasm for psychiatry may be engendered in experiences such as the management of treatment-resistant schizophrenia, rather than the topics that will be more useful for the Foundation Years. However, undergraduate experience is not the only factor of importance in career choice. It has been shown that only 46% of doctors who qualified in the United Kingdom who finally specialised in psychiatry had it as their first choice career one year after qualification [16]. Therefore, postgraduate experience and personal circumstances must play an important role.

A limitation of the method of collecting data was that students were only surveyed if they attended the lecture. This excluded more of the 5^th ^years as more did not attend and is a possible source of bias. The ideal study design would have been a cohort of students questioned before the 4th year of training and after completing the 5^th ^year. However, this was not possible as for reasons of confidentiality the surveys were anonymous. In addition, this survey was carried out in one medical school and differences in the emphasis and implementation of teaching programmes in other medical schools may yield different results.

## Conclusion

Two ways that medical students believe their teaching can be made more relevant to their future careers are highlighted in this study. Firstly, there is a need to focus on scenarios which students will commonly encounter in their Foundation Years (initial years of employment). Secondly, psychiatry should be better integrated into the overall curriculum, with the opportunity for teaching in different settings. It has previously been suggested that teaching in psychiatry that is relevant to students' likely future clinical experience can occur in primary care or general hospitals [17]. In terms of curriculum development, this study illustrates the need to listen to what students believe they should learn but that this needs to be balanced against the necessity of teaching the fundamentals and principles of a speciality.

## Competing interests

FO is the Head of the Department of Psychiatry at the University of Birmingham.

## Authors' contributions

CO conceived and carried out the study. FO provided advice on the design of the study. Both authors contributed to and approved the final manuscript.

## Pre-publication history

The pre-publication history for this paper can be accessed here:



## Supplementary Material

Additional file 1 Medical student questionnaire – 4th. A copy of the questionnaire distributed to the medical students. Click here for file
