# Supplementary figures and images for: Medical students' views about an undergraduate curriculum in psychiatry before and after clinical placements
Source: BMC Med Educ. 2008 Apr 25;8:26. doi: 10.1186/1472-6920-8-26 (PMC2383892; doi:10.1186/1472-6920-8-26)

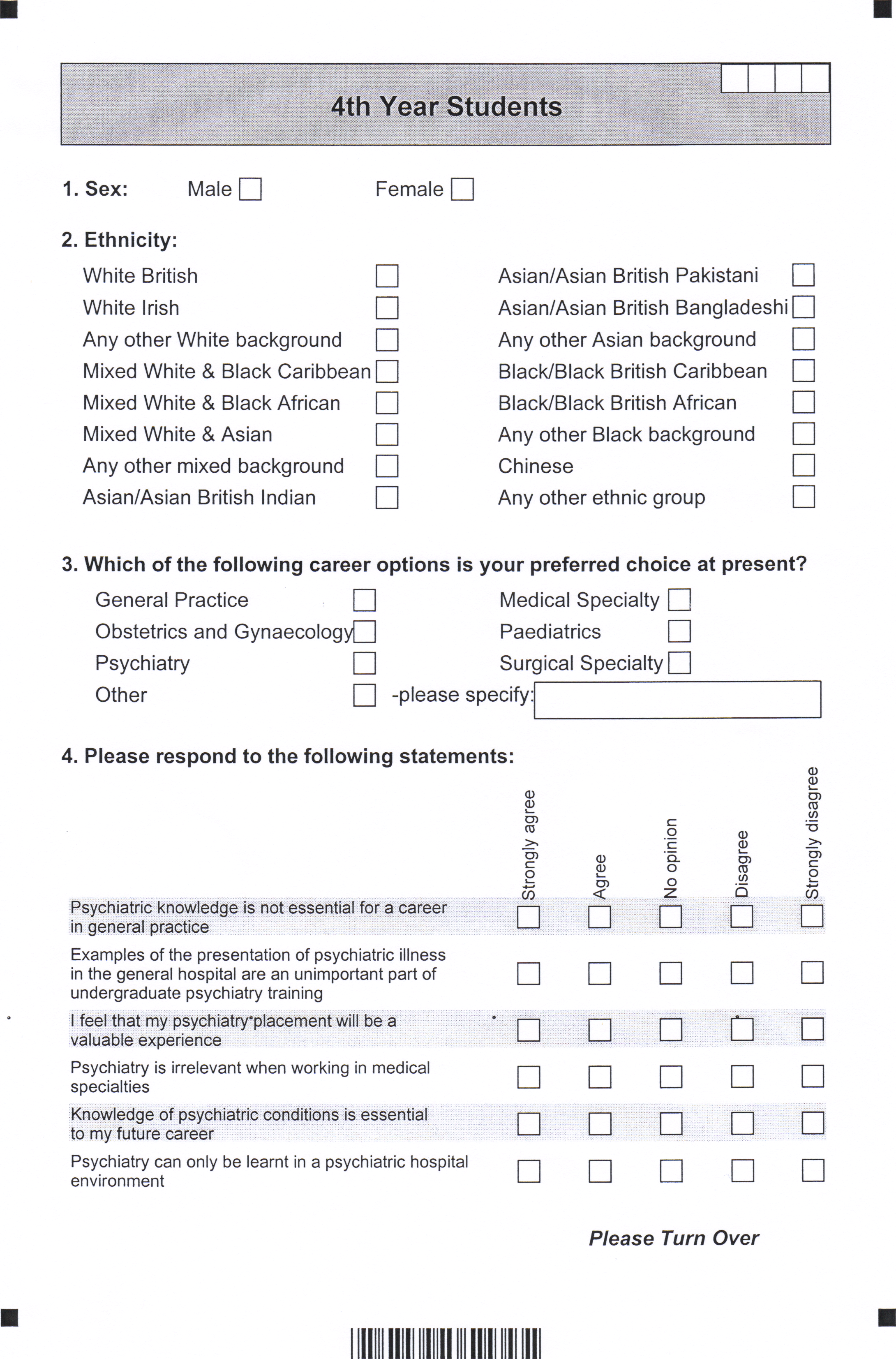


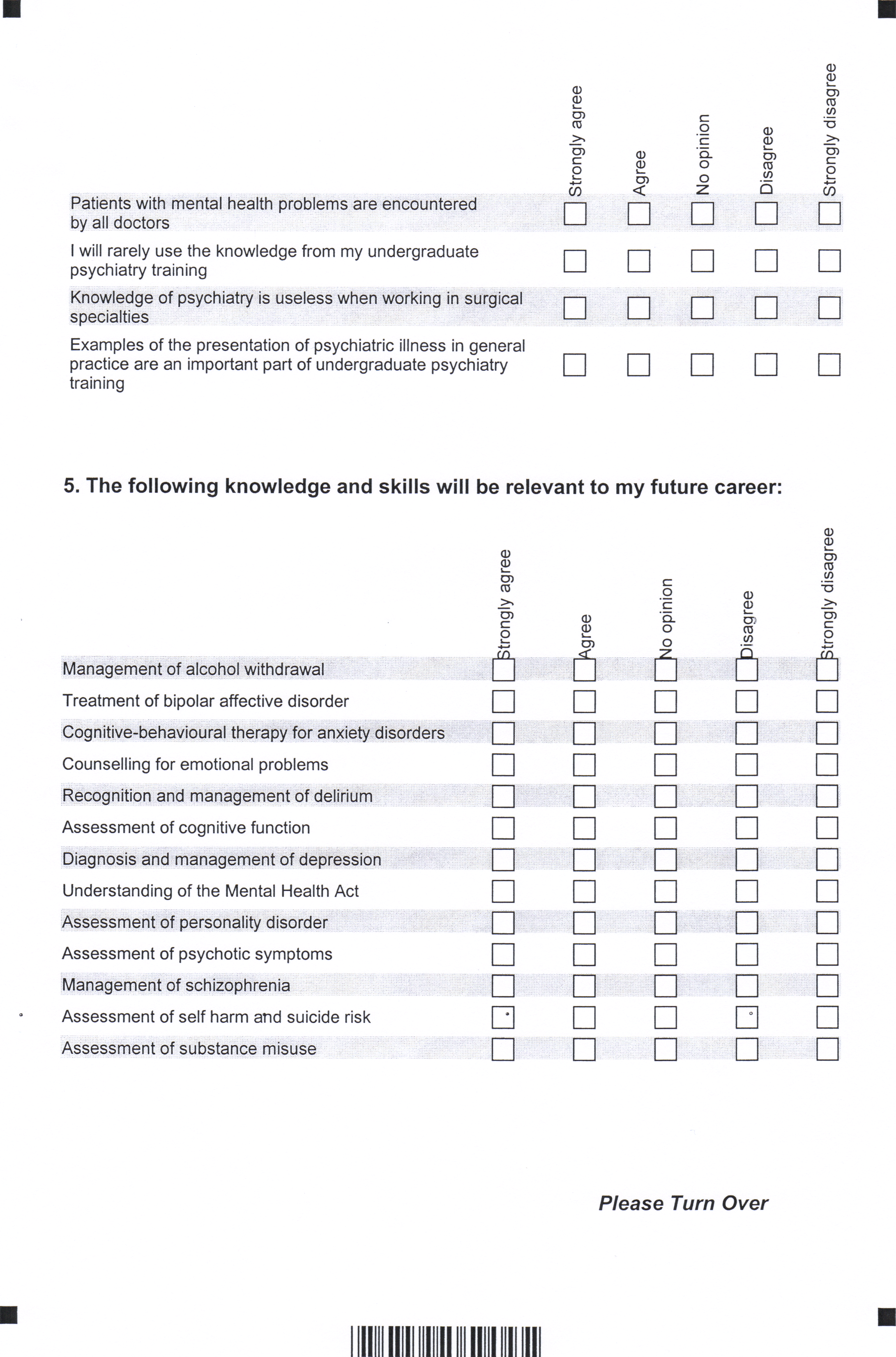


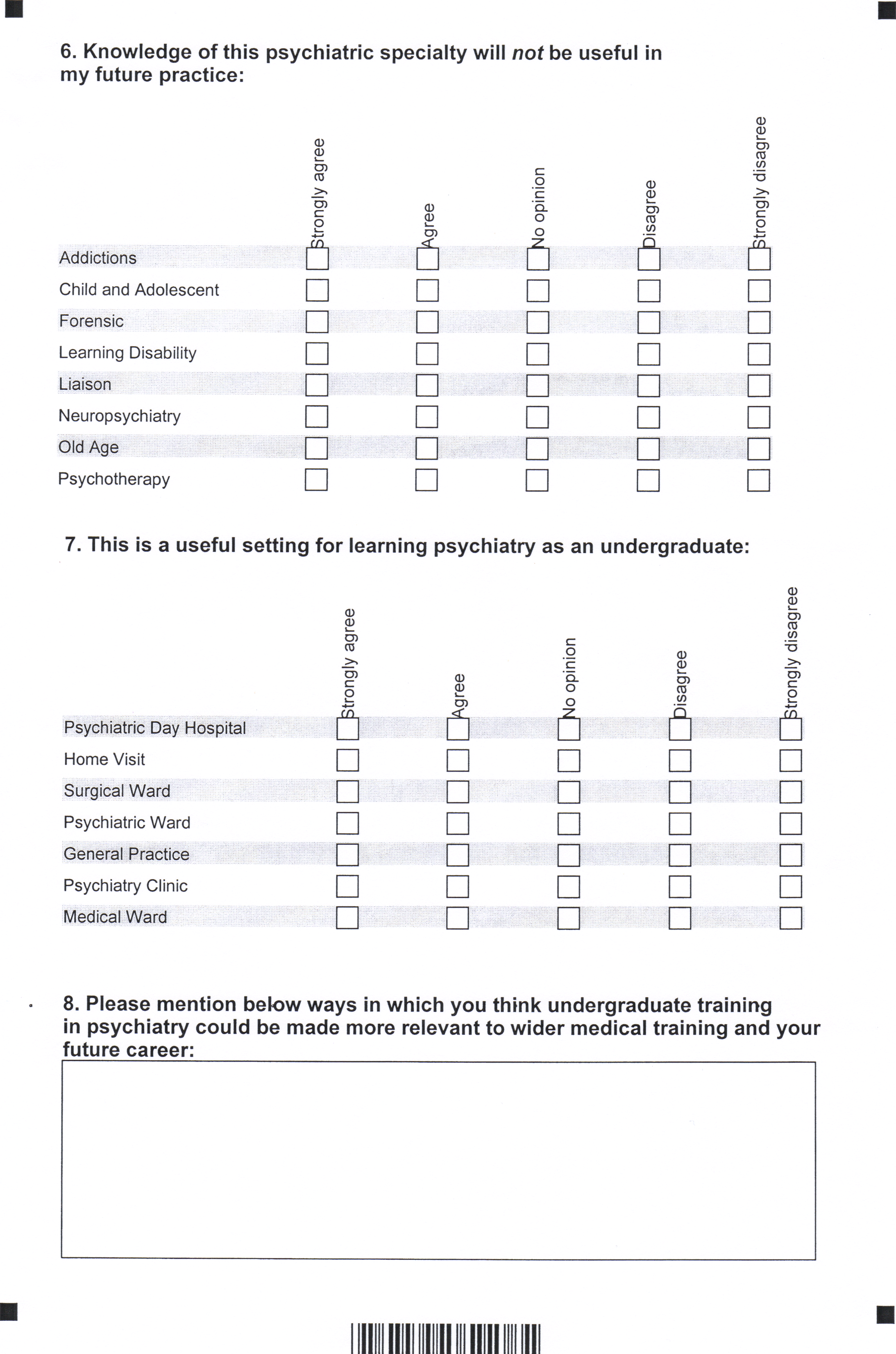

Supplement: Additional file 1 — Medical student questionnaire – 4th. A copy of the questionnaire distributed to the medical students. [file 1472-6920-8-26-S1.doc]
